# Targeted nanomedicines for the treatment of *Helicobacter pylori* infection

**DOI:** 10.1016/j.mtbio.2025.101820

**Published:** 2025-04-30

**Authors:** Shi Wang, Hao Ding, Longsong Li, Ruifang Zhao, Ningli Chai

**Affiliations:** a Department of Gastroenterology, The First Medical Center of Chinese PLA General Hospital, Beijing, 100853, PR China; b Graduate School of the People's Liberation Army (PLA) General Hospital, Beijing, 100853, PR China; c CAS Key Laboratory for Biomedical Effects of Nanomaterials and Nanosafety, CAS Center for Excellence in Nanoscience, National Center for Nanoscience and Technology, Beijing, 100190, PR China

**Keywords:** *Helicobacter pylori*, Nanomedicine, Antibacterial nanomaterials, Drug delivery, Antibiotic resistance

## Abstract

*Helicobacter pylori* is a significant risk factor for gastric cancer, making its elimination crucial for prevention. This review explores the application of nanotechnology in enhancing treatment efficacy against *H. pylori* and addressing antibiotic resistance. Nanomaterials are utilized as drug carriers to protect pharmaceuticals from the acidic gastric environment and enable controlled release. These materials can be specifically engineered to target *H. pylori* surface molecules, thereby improving antimicrobial efficacy. Enhanced drug penetration is achieved by optimizing the surface properties and molecular architecture of nanomaterials. Additional strategies include targeting *H. pylori* virulence factors, such as urease and vacuolating cytotoxin A, as well as inhibiting bacterial proliferation through reactive oxygen species-based therapies. Moreover, the ability of nanomaterials to effectively modulate the microbiota and inhibit pathogens has shown great potential in the application of combating *H. pylori*. By focusing on specific invasion mechanisms and metabolic pathways, nanotechnology offers significant promise for advancing treatments against *H. pylori*. Despite its potential, several challenges remain, including high drug development costs, difficulties in precise targeting, and adverse effects associated with certain approaches, such as photothermal therapy. Ongoing optimization of nanotechnological strategies is anticipated to address these challenges, thereby facilitating the development of more effective and targeted therapies for *H. pylori* infection.

## Graphical abstract

**Figure:**
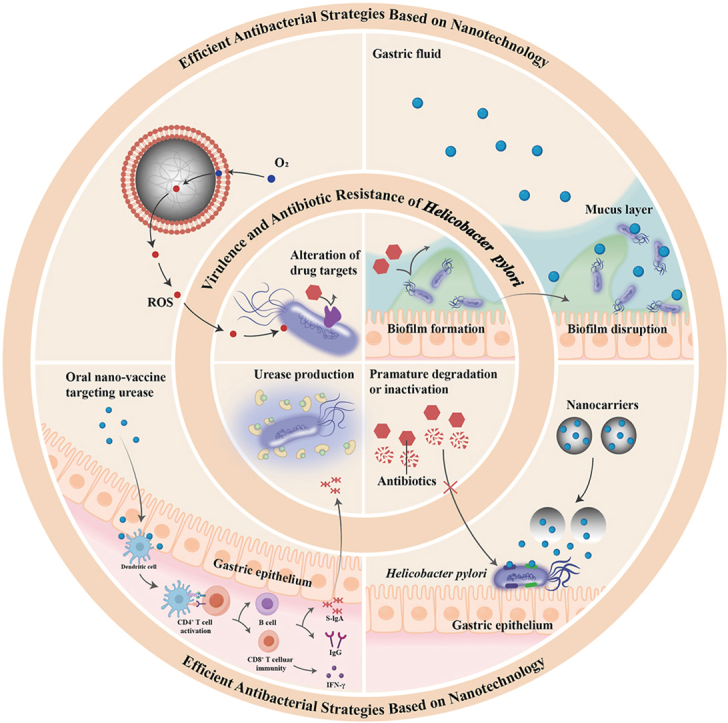

## 1 Introduction

Gastric cancer ranks as the fifth leading cause of cancer-related mortality worldwide, with over 950,000 new cases reported annually [1]. A majority of these cases are attributed to *Helicobacter pylori* infection, classifying it as a cancer associated with chronic inflammation [2]. *H. pylori*, a Gram-negative bacterium, colonizes the gastric mucus and epithelium, inducing inflammation in the host's gastric epithelial cells. This inflammation contributes to the development of various gastric diseases, including peptic ulcer disease, gastric adenocarcinoma, atrophic gastritis, and mucosa-associated lymphoid tissue lymphoma [3,4]. Current epidemiological guidelines emphasize the importance of widespread screening and treatment for *H. pylori* infection to facilitate the healing of inflammation and prevent the progression of mucosal and genetic damage caused by *H. pylori* [5,6]. Thus, screening and eradicating *H. pylori* infection are critical measures for preventing gastric diseases.

The eradication regimen for *H. pylori* is based on a combination of potent acid suppressants and antibiotics [6]. The first-line treatment typically involves proton pump inhibitor (PPI)-based triple therapy (PPI-TT), which includes antibiotics such as clarithromycin, amoxicillin, and metronidazole, or, in cases of higher resistance, levofloxacin. Antibiotic resistance is the most significant factor contributing to the failure of PPI-TT [7]. Treatment failure often leads to the development of resistance to multiple antibiotics used in first-line regimens, thereby complicating and increasing the cost of subsequent treatments [8,9]. A European registry study on *H. pylori* treatment analyzed 2852 treatment-naive patients and reported resistance rates of 25 %, 30 %, and 20 % for clarithromycin, metronidazole, and levofloxacin, respectively [10]. The increasing prevalence of resistance poses a significant challenge to the clinical management of *H. pylori* infection. The World Health Organization has classified clarithromycin-resistant *H. pylori* infection as a high-threat, community-acquired infection [11]. Given the rising resistance rates, there is an urgent need to develop innovative therapeutic strategies to manage *H. pylori* infection effectively.

Nanomaterials possess a high surface area-to-volume ratio, along with tunable optical, electronic, magnetic, biological, and chemical properties. These features are being integrated into next-generation drug delivery systems, contrast agents, and diagnostic devices. Some of these applications are currently under clinical investigation or have already received approval from the U.S. Food and Drug Administration (FDA) for human use, demonstrating excellent biocompatibility [12]. Compared to traditional antibiotics, nanomaterials enable rational design, including size control, surface modification, alterations in crystallinity, and stimuli-responsive functionalization, facilitating unique interactions with bacterial cells [13]. Nanomaterials can function not only as nanocarriers for antimicrobial agents, effectively delivering drugs to target bacterial cells, but also exhibit intrinsic therapeutic effects by integrating multiple antimicrobial strategies, thereby demonstrating multi-modal bactericidal potential. Furthermore, nanomedicines offer prolonged, resistance-independent antimicrobial effects, effectively preventing the emergence of antimicrobial resistance [14]. Nanomedicine presents significant potential for the treatment of *H. pylori* infection.

This article summarizes the virulence mechanisms and antibiotic resistance characteristics of *H. pylori*, and provides a detailed review of nanomedicines for targeted therapy, categorizing them based on their design to target *H. pylori* ‘s virulence mechanisms and resistance traits. It provides valuable insights and guidance for researchers striving to develop more comprehensive and effective antimicrobial platforms against *H. pylori*.

## 2 Virulence and antibiotic resistance of *H. pylori*

**Fig. 1:**
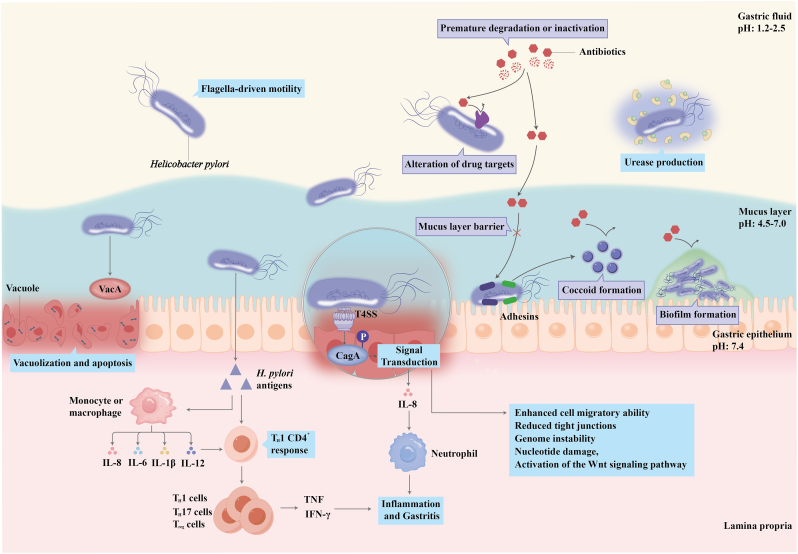
Pathogenic mechanisms and antibiotic resistance of *Helicobacter pylori.* The pathogenic mechanisms of *H. pylori* are outlined in blue. *H. pylori* colonizes the gastric mucosa through flagellar motility, urease production, and adhesion. It secretes vacuolating cytotoxin A (VacA), causing vacuolation and apoptosis in gastric epithelial cells. The Type IV secretion system (T4SS) injects CagA antigen into host cells, disrupting intracellular signaling pathways, promoting the expression and release of interleukin-8 (IL-8), and recruiting neutrophils, thereby contributing to the development of gastritis. *H. pylori*-specific antibodies trigger host immune responses, including activation of macrophages derived from monocytes and complex interactions among TH1, TH17, and regulatory T cells (Tregs). Biological attributes of resistance in *H. pylori* are outlined in purple. The acidic gastric environment leads to the premature degradation of antibiotics. Genetic mutations alter drug targets, reducing efficacy. The mucus layer and biofilm formation act as barriers to drug penetration. Additionally, the coccoid dormant state of *H. pylori* greatly enhances its antibiotic resistance. (For interpretation of the references to colour in this figure legend, the reader is referred to the Web version of this article.)

*H. pylori* is highly adapted to colonizing the gastric epithelium and thriving in the acidic environment of the stomach. Its colonization primarily depends on flagella-driven motility, which enables the bacterium to penetrate the mucus layer and reach epithelial cells [15]. Additionally, *H. pylori* produces urease, an enzyme that hydrolyzes urea into ammonia and carbon dioxide, thereby neutralizing gastric acid and facilitating its survival in the acidic stomach [16]. Furthermore, *H. pylori* adheres to gastric epithelial cells through surface molecules, such as blood group antigen-binding adhesin (BabA) and sialic acid-binding adhesin (SabA), which promote stable colonization despite the continuous turnover of epithelial cells and mucus [17] (Fig. 1).

Several factors drive the pathogenicity of *H. pylori*, the most significant of which is the *cag* pathogenicity island, which encodes a type IV secretion system (T4SS) [18]. This system enables *H. pylori* to inject virulence factors, including the cytotoxin-associated gene A (CagA) antigen, directly into host cells. Once inside host cells, CagA undergoes tyrosine phosphorylation by cellular kinases [19], a modification that allows it to interact with multiple target molecules within the host cell. This interaction promotes enhanced cell motility, reduced cellular tight junctions, genome instability, nucleotide damage, and activation of the Wnt signaling pathway, which is implicated in local neoplasia formation [20]. In addition, other bacterial components can be translocated into host cells via the T4SS, including the heptose-containing lipopolysaccharides (LPS) core precursors [21,22], peptidoglycan fragments [23], and bacterial DNA [24]. These molecules interact with intracellular target molecules, thereby influencing intracellular signaling pathways. Many of the affected pathways converge on the activation of nuclear factor (NF)-*κ*B, leading to increased expression and release of interleukin-8 (IL-8) and other chemokines and cytokines [25,26,27]. IL-8, as a potent neutrophil chemoattractant, plays a critical role in the accumulation of neutrophils within the gastric mucosa, a defining feature of chronic active gastritis [28,29]. Another important virulence factor is Vacuolating cytotoxin A (VacA), a toxin with oligomeric autotransporter properties capable of forming anion-selective membrane channels [30] (Fig. 1). VacA exerts various effects on cells, including the formation of large intracellular vacuoles originating from late endosomes, the promotion of apoptotic cell death (linked to mitochondrial membrane disruption) or necrosis, and the induction of autophagy [31,32,33].

The persistent infection of *H. pylori* is largely attributed to its ability to mediate immune evasion. The flagella and LPS of *H. pylori* differ significantly from those of other Gram-negative bacteria, allowing it to evade recognition by pattern recognition receptors, such as TLR5 and TLR4, which are typically responsible for detecting bacterial intruders [34,35]. Moreover, *H. pylori* heptose metabolites, such as ADP-heptose, downregulate the expression of HLA-II genes, thereby suppressing the antigen-presenting function of macrophages and impairing subsequent T cell responses [36]. In addition, dendritic cells in contact with the bacteria can undergo reprogramming, such as inducing IL-18 production, which promotes the differentiation of T cells into regulatory T cells, ultimately inhibiting immune activation [37].

Therefore, the colonization of gastric mucosa by *H. pylori* triggers a pro-inflammatory response in gastric epithelial cells, promoting the recruitment of immune cells to the submucosa [38]. This immune-mediated inflammatory response leads to chronic active gastritis. Persistent inflammation of the gastric mucosa is recognized as a key driver of gastric atrophy and, ultimately, gastric cancer, as described in the Correa cascade. This cascade outlines a multistage, multifactorial progression that begins with superficial gastritis and advances through atrophic gastritis, intestinal metaplasia, and dysplasia, culminating in gastric adenocarcinoma [39].

*H. pylori* gastritis is an infectious disease that requires treatment in all infected adults with clinical symptoms or complications, and preventive treatment in asymptomatic individuals if there is a risk of developing complications [40,41,42]. Initially, PPI-TT was the standard treatment regimen, combining a PPI with clarithromycin, amoxicillin, or metronidazole, achieving an eradication rate of over 90 % with a 7-day course [43]. However, as clarithromycin resistance has risen globally, the effectiveness of triple therapy has declined. According to international treatment guidelines, in regions where clarithromycin resistance exceeds 15 %, clarithromycin-based triple therapy is no longer recommended as a first-line option [7]. As a result, bismuth-based quadruple therapy (BiQT) has emerged as an effective alternative, particularly in areas with high clarithromycin resistance [41]. Bismuth enhances the efficacy of metronidazole, so even in cases of metronidazole resistance, the effectiveness of quadruple therapy remains largely unaffected [44,45,46]. Consequently, BiQT is considered one of the most effective and widely adopted treatment regimens globally.

To address high resistance rates to clarithromycin and metronidazole, concomitant therapy and hybrid therapy were introduced. While these regimens show promising eradication rates [47,48], their complex administration may impact patient adherence and potentially promote further antibiotic resistance [49,50]. Both tetracycline and amoxicillin exhibit low resistance rates (typically less than 1 %), making them core components of many treatment regimens [7,46]. In contrast, rifabutin and furazolidone are primarily reserved for rescue therapy in cases of multiple treatment failures. Although rifabutin has a low resistance rate, its rare but serious side effect of bone marrow toxicity, along with its importance in treating other critical infections, limits its routine use [9,41,51]. Amid the rising threat of antibiotic resistance, a deeper understanding of the mechanisms driving bacterial resistance is essential for developing more effective treatment strategies and crafting precise, personalized therapies.

The antibiotic resistance of *H. pylori* poses a significant global threat to human health. The factors contributing to this resistance primarily include chromosomally encoded mutations and physiological changes, such as impaired regulation of drug uptake and efflux, along with the formation of biofilm and coccoid forms [52] (Fig. 1). Gene mutations typically occur in genes related to antibiotic targets, altering the binding sites of antibiotics or reducing their activity within the cell. These mutations drive resistance to various classes of drugs, contributing to a cumulative multidrug resistance profile [53,54]. Nevertheless, other mechanisms of multidrug resistance also play a role. These mechanisms include the upregulation of efflux pump systems, which reduce the accumulation of antibiotics within the cell [55,56,57]. Additionally, *H. pylori* can form biofilm in vivo, with the biofilm matrix composed of extracellular polymeric substances that create a structured microbial community [52]. This matrix acts as an effective nonspecific barrier, impeding drug penetration and reducing antimicrobial activity. Furthermore, nutrient and oxygen deprivation within the biofilm leads to decreased bacterial metabolic activity, further enhancing tolerance to antibiotics [58]. The presence of the biofilm also facilitates horizontal gene transfer (HGT) and overexpression of efflux pumps associated with drug resistance [59,60,61]. Consequently, *H. pylori* residing within biofilm is more likely to persist long-term, contributing to chronic and recurrent infections while evolving antibiotic resistance [57,62,63,64].

Under unfavorable environmental conditions, *H. pylori* can transition into a coccoid form, a dormant state characterized by reduced nutrient requirements, decreased metabolic activity, and enhanced resistance to harsh environments. This transformation involves morphological and surface modifications, along with ultrastructural and metabolic changes, which reduce the exposure of drug targets, limit antibiotic penetration, and increase antibiotic efflux [65,66,67]. Moreover, the coccoid form lacks metabolic byproducts that stimulate immune factors such as NF-*κ*B and IL-8 [68]. During this transition, the expression of many bacterial surface antigens is altered [69], further enhancing immune evasion. Consequently, significantly higher minimum inhibitory concentrations (MICs) of antibiotics are required to achieve bactericidal effects against coccoid *H. pylori* [65,70,71]. The highly acidic environment in the stomach, combined with the abundance of digestive enzymes, can also lead to premature degradation or inactivation of antibiotics. Moreover, the dense mucus layer on the gastric epithelial surface significantly hinders the efficiency of drug delivery to the mucosa [72]. When the effective concentration of the drug fails to reach bactericidal levels, *H. pylori* remains exposed to suboptimal antibiotic concentrations, further increasing the selective pressure for resistance and accelerating the emergence of resistant bacterial strains [73].

The growing antibiotic resistance of *H. pylori* remains a critical challenge, as combination antibiotic therapies have not fundamentally resolved the issue of resistance and may even contribute to its spread or cause serious adverse effects. Therefore, developing innovative treatment strategies targeting the invasive and resistance mechanisms of *H. pylori* is essential for improving therapeutic outcomes and addressing recurrent infections.

## 3 The application of nanomedicines in targeting *H. pylori* infection

The widespread global infection of *H. pylori* and the increasing dissemination of antibiotic resistance have driven the development of novel therapeutic approaches for bacterial infections. Nanoparticles, with their high surface area-to-volume ratio, offer versatile modification capabilities that can be utilized to design innovative treatment strategies. Nanotechnology facilitates the integration of multiple antimicrobial mechanisms into a single platform, significantly enhancing antibacterial efficacy while simultaneously delaying the emergence of resistance. This article reviews recent advancements in nanomedicines for the treatment of *H. pylori* and classifies these nanomedicines based on their design to target specific invasive or resistance mechanisms of *H. pylori*.

### 3.1 Optimization of nanocarriers: high-efficiency encapsulation and controlled release

#### 3.1.1 High-efficiency encapsulation of nanodrugs

**Fig. 2:**
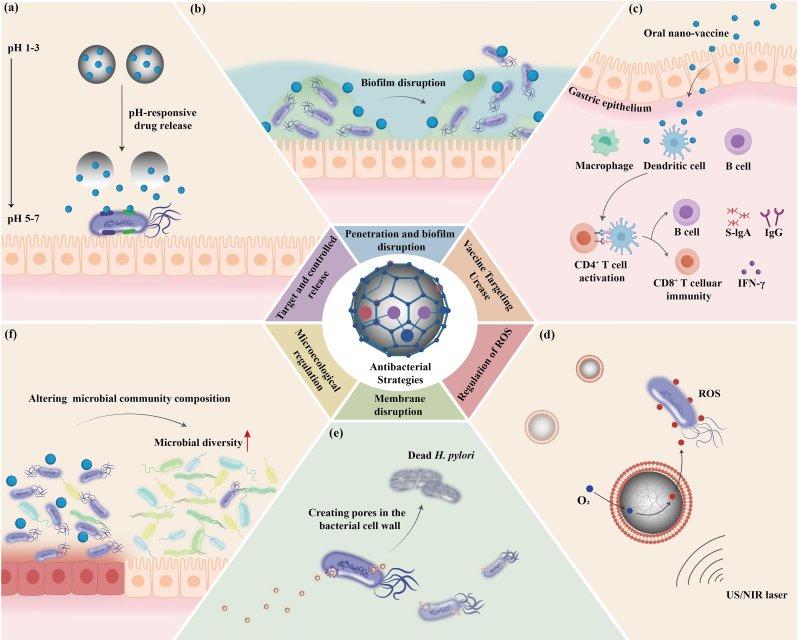
Antibacterial strategies of nanomedicines targeting *Helicobacter pylori.* (a) Nanoparticles precisely target *H. pylori* and enable controlled release of antibacterial agents. (b) Nanomedicines disrupt biofilm and eradicate *H. pylori*. (c) Oral nano-vaccines induce immune responses in the gastric mucosa. (d) Nanoparticles release reactive oxygen species (ROS) in response to light or sound stimuli, interfering with *H. pylori* ‘s normal metabolism. (e) Metal nanoparticles mediate direct bactericidal effects by disrupting bacterial cell membranes. (f) Nanoparticles can alter the composition of microbial communities, promote the growth of beneficial microorganisms, and antagonize *H. pylori*, thereby enhancing microbial diversity.

The highly acidic conditions in the stomach, combined with the abundance of digestive enzymes, present significant challenges to the bioactivity of drugs [74,75]. *H. pylori* colonizes the gastric mucosal epithelium [76], and oral drugs are often partially inactivated or degraded before reaching the site of bacterial colonization. This results in insufficient local drug concentrations, thereby increasing the selective pressure for resistant bacteria. Simply increasing the dosage or prolonging the treatment duration may lead to severe side effects and further promote antibiotic resistance. Consequently, the development of efficient nano-delivery systems to enhance drug utilization is crucial for improving therapeutic success and, to some extent, delaying the progression of resistance (Fig. 2a).

A primary consideration in these systems is ensuring drug stability during transport. HP55/Poly (n-butyl cyanoacrylate) (PBCA) nanoparticles, developed by Liu et al. [77], demonstrate significant protective effects as carriers resistant to acidic environments and proteolytic enzymes. These nanoparticles effectively prevent the rapid degradation of antigens in simulated gastric fluid (pH 1.2) and provide sustained protection in simulated intestinal fluid (pH 7.4). This protective mechanism preserves antigen activity by delaying degradation and ensures controlled release, enabling antigen delivery at the right time and location, thereby greatly enhancing the vaccine's efficacy.

The high-efficiency encapsulation of drugs targeting *H. pylori* by nanomaterials protects the drug's activity in complex acidic environments and addresses the issue of poor solubility for certain medicines, further enhancing their therapeutic efficacy. Obonyo et al. [78] designed a liposomal nanoparticle (LipoLLA) that utilizes the amphiphilic properties of liposomes to encapsulate the antimicrobial fatty acid linolenic acid (LLA) within its hydrophobic region. This design prevents the oxidation and esterification of the fatty acid and addresses the poor solubility of lipid-based drugs, enabling stable and efficient delivery to target sites within the body. Upon reaching the site of infections, the liposomes fuse with bacterial membranes, directly releasing the encapsulated fatty acids into the bacterial membrane. This process disrupts the bacterial membrane structure, leading to cell death. The membrane fusion mechanism of liposomes reduces the interaction of the drug with intracellular bacterial targets, thereby lowering the likelihood of resistance development through conventional mechanisms. Moreover, LipoLLA has demonstrated significant bactericidal efficacy against *H. pylori* in its dormant coccoid form. In vitro results showed that LipoLLA achieved a 99 % kill rate, and *H. pylori* treated with LipoLLA did not develop drug resistance within 10 days, whereas resistance to LLA alone emerged by day 3 [78].

#### 3.1.2 Controlled release of nanodrugs

Additionally, nanocarriers can be engineered for controlled, on-demand drug release triggered by specific environmental factors, such as pH. This targeted release mechanism ensures that antibiotics are accurately delivered to the infection site, increasing the effective drug concentration, optimizing therapeutic outcomes, and minimizing the formation of selective pressure environments that promote the development of resistant bacteria due to insufficient drug concentrations.

Jing et al. [79] developed amoxicillin-loaded UCCs-2/TPP nanoparticles that leverage a pH-responsive release mechanism. These nanoparticles release their drug payload in the low-pH environment surrounding *H. pylori* colonies. However, the loaded amoxicillin exhibited burst release in gastric fluid (pH 1.2), with approximately 50 % released within 2 h and around 65 % released over 4 h near the mucosal layer (pH 6.0). This rapid release in the stomach may result in partial drug depletion before reaching the infection site, potentially reducing therapeutic efficacy. Furthermore, excessive initial release could increase systemic drug exposure, raising the risk of side effects while diminishing sustained drug availability required for prolonged therapeutic effects, ultimately compromising overall treatment efficacy.

To achieve sustained drug release, Lin et al. [80] developed genipin-crosslinked fucose-chitosan/heparin nanoparticles (genipin-FCS/Hep NPs) encapsulating low molecular weight amoxicillin. Genipin, a naturally occurring crosslinker, was utilized as a noncytotoxic alternative to glutaraldehyde. The nanoparticles were fabricated using an emulsion method, which enhanced their structural stability, protected the drug from degradation, and reduced its release rate. In gastric fluid (pH 1.2), the nanoparticles released 17.1 ± 1.9 % of amoxicillin over 120 min, while only minimal amounts were released near the mucosal layer (pH 6.0), successfully achieving sustained drug release. Additionally, the chitosan nanoparticles increased adhesion to gastric mucosa through electrostatic interactions, further prolonging drug retention.

*H. pylori* infection is a dynamic process regulated by both time and space. In the design of nanoparticle drug delivery systems, traditional multi-functional component synergistic strategies lack spatiotemporal control, making it challenging to achieve optimal efficacy in the complex gastric environment, which results in suboptimal eradication outcomes [81]. In contrast to synergistic strategies, Zhang et al. [81] developed a nanodrug delivery system, D@SMART, which utilizes depletion interactions to simultaneously regulate the self-assembly of multi-compartment metal-organic frameworks (MOFs) to form well-organized hierarchical structures, termed SMART technology (Surfactant-guided Heterogeneous MOF Architecture). These heterogeneous layered nanocarriers maintain their integrity in the stomach, protecting multiple components until they reach the target site, enabling spatiotemporal drug release to treat *H. pylori* precisely. The spiny crown layer contains alginate to interfere with *H. pylori* adhesion, while dietary polyphenol tannic acid, with strong anti-*H. pylori* activity, is encapsulated in the outer shell. Resveratrol, a natural plant antibiotic polyphenol with potent antioxidant properties that can inhibit pro-inflammatory signaling pathways, is encapsulated in the core layer of the SMART system. In a mouse infection model, D@SMART effectively eradicated *H. pylori*, reduced inflammation, and repaired damaged gastric mucosa. Unlike conventional antibiotic treatments, D@SMART also helped prevent intestinal microbiota imbalance, providing a more precise, effective, and safer approach for clinical treatment of *H. pylori* infection.

#### 3.1.3 Precision targeting of nanodrugs

The development of targeted therapies against *H. pylori* has primarily focused on exploiting specific receptors on bacterial surfaces [79,82]. For example, Jing et al. [79] developed UCCs-2/TPP nanoparticles that achieved selective targeting and eradication of *H. pylori* by leveraging specific interactions between the urea groups of UCCs-2 and the urease channel protein (UreI) on the surface of *H. pylori*. The selectivity of UCCs-2/TPP nanoparticles toward *H. pylori* was further confirmed by introducing the competitive substrate urea, which demonstrated their targeted behavior. Similarly, Lin et al. [80] developed genipin-FCS/Hep NPs that utilized fucose, a component explicitly recognized by the *H. pylori* surface lectin, enabling precise drug release at the site of infection. This targeted drug release mechanism was validated in animal studies, where it effectively acted on *H. pylori* and reduced gastric inflammation associated with the infection.

Inspired by the natural interactions and adhesion between pathogens and hosts, Angsantikul et al. [83] coated the surface of nanoparticles with gastric epithelial cell membranes, creating biomimetic nanoparticles (AGS-NPs) loaded with clarithromycin. This coating allowed AGS-NPs to carry cell surface antigens utilized by *H. pylori* for attachment and colonization, thereby conferring targeted adhesion to the bacteria. In both in vitro and animal model studies, AGS-NPs exhibited significantly stronger antibacterial effects compared to free clarithromycin, with excellent biocompatibility.

Aptamers have gained significant attention as highly specific binding agents for various ligands [84]. Compared to traditional antibodies, aptamers exhibit comparable binding affinity and specificity, offering advantages such as high stability, the ability for large-scale chemical synthesis, and low immunogenicity [85]. Wang et al. [86] developed an antibody-independent biosensing platform for the specific diagnosis of *H. pylori*. The platform's first module consists of calcium-doped superparamagnetic nanoparticles functionalized with an *H. pylori*-specific aptamer, enabling the selective capture of *H. pylori* cells. This system achieved over 99.0 % capture efficiency from mixed bacterial communities, demonstrating the Hp1 aptamer's high specificity and reduced nonspecific binding. This study introduces a promising approach where aptamer-based detection strategies could be extended to design targeted nanomedicine, improving drug delivery efficiency and minimizing disruption to gastrointestinal microbiota.

Overall, functionalized nanomedicine carriers protect the bioactivity of drugs and slow their release. They can also be engineered for controlled release in response to specific environmental factors, such as pH, ensuring precise delivery of antibiotics to the site of infection and thereby optimizing therapeutic efficacy. Combined with precise targeting strategies that exploit specific receptors on *H. pylori*, these systems further enhance bactericidal effects while minimizing drug toxicity. In addition to improving bacterial eradication efficiency, these systems reduce the selective pressure exerted by low concentrations of antibiotics on *H. pylori*, thereby mitigating the development of antibiotic resistance to some extent. However, the use of nanocarriers for effective drug delivery remains largely limited to the bactericidal mechanisms of antibiotics. While this approach can enhance the killing efficiency against resistant bacteria, it does not prevent the spread of resistance. Therefore, leveraging nanotechnology to develop multi-mechanism antimicrobial strategies is crucial. Such strategies could not only improve bacterial eradication but also help avoid the emergence of resistance.

### 3.2 Overcoming physical barriers: mucus penetration and biofilm disruption

*H. pylori* colonizes the gastric mucosal epithelium, which is covered by a dense mucus layer [76]. This layer, composed of approximately 95 % water and 5 % negatively charged mucins, is a barrier that significantly hinders drug access to the infection site [87]. Additionally, *H. pylori* forms biofilm within the mucus layer on the gastric mucosal surface [88]. Biofilm is a three-dimensional structure formed by microorganisms and extracellular polymeric substances (such as polysaccharides, proteins, and nucleic acids). These microorganisms aggregate on a surface and are encased by the extracellular polymers they secrete, creating a protective barrier. The biofilm's compact, negatively charged extracellular polymeric matrix is a secondary barrier, further impeding drug penetration and significantly increasing the bacteria's antibiotic resistance [58,89]. These dual barriers present critical challenges in developing effective strategies for eradicating *H. pylori*. Therefore, antimicrobial strategies based on nanotechnology to penetrate the mucus layer and disrupt biofilm represent a crucial approach to improving the eradication efficiency of *H. pylori* (Fig. 2b).

#### 3.2.1 Nanodrug penetration through the mucus barrier

Modifying nanoparticles to regulate their surface characteristics and enhance their diffusion through the mucus layer is an effective strategy for improving drug delivery. Polyethylene glycol (PEG) is a hydrophilic and non-ionic polymer that reduces the adhesion of nanoparticles to the mucin fibers within the mucus mesh, enabling nanoparticles to rapidly penetrate the low-viscosity interstitial fluid between mucin fibers [90,91,92]. Zhang et al. [93] designed self-assembling nanoparticles that bind to antigens and cell-penetrating peptides (CPP) via electrostatic self-assembly and are coated with a “mucus-inert” PEG derivative. The PEG-coated nanoparticles exhibit hydrophilic and slightly negatively charged surface characteristics, which minimize their interaction with gastrointestinal mucus and enhance their ability to penetrate the mucus layer effectively. Upon successful penetration, the nanoparticles expose a core enriched with CPP and R12 (poly-L-arginine), which act as cell-penetrating peptides to facilitate efficient transmembrane absorption via both intracellular and paracellular pathways. This targeted delivery triggers specific immune responses, effectively eliminating *H. pylori* and alleviating gastric inflammation caused by the infection.

The use of external forces can also facilitate the penetration of nanomedicines through the mucus layer, enhancing drug delivery efficiency. Superparamagnetic iron oxide (SPIO) nanoparticles are a widely favored metallic material extensively applied in drug/gene delivery, magnetic separation, and magnetic hyperthermia for cancer treatment [94,95,96]. Yang et al. [97] developed a chitosan/polyacrylic acid-based particle system co-loaded with SPIO nanoparticles and amoxicillin (SPIO/AMO@PAA/CHI) for the treatment of *H. pylori* infection. Leveraging the adhesive properties of chitosan, the iron oxide nanoparticles initially adhere to the gastric mucus layer. Assisted by an external magnetic field, the nanoparticles successfully penetrate the mucus, overcoming the first barrier to effective drug delivery. Furthermore, applying the magnetic field significantly prolongs the retention time of the nanoparticles in the stomach. Subsequently, polyacrylic acid competes with amoxicillin for binding to chitosan, enabling a rapid and sustained release of amoxicillin at the gastric epithelial surface, directly targeting *H. pylori*. This targeted approach significantly reduces both the drug dosage and treatment duration required for the eradication of *H. pylori*.

#### 3.2.2 Nanodrug disruption of bacterial biofilms

A biofilm is a community of bacteria that aggregate within a self-produced extracellular polymeric substance (EPS), consisting of exopolysaccharides, proteins, extracellular bacterial DNA, and enzymes. This biofilm creates a protective environment where planktonic bacteria can survive, reproduce, and communicate through quorum sensing (QS) [98,99,100,101]. QS is a mechanism by which bacteria communicate through signaling molecules, coordinating the structure and function of biofilm. Inhibiting bacterial QS has been identified as an effective strategy for combating biofilm formation [102]. Gopalakrishnan et al. [103] designed silver nanoparticles stabilized by N-acyl homoserine lactonase (AiiAAg nanoparticles) that inhibit biofilm formation by degrading QS signaling molecules. Additionally, these nanoparticles suppress urease activity in *H. pylori*, further enhancing their antimicrobial efficacy.

Rhamnolipid (RHL) is an anionic biosurfactant secreted by *Pseudomonas aeruginosa* that inhibits biofilm formation by preventing bacterial adhesion. It also significantly disrupts mature biofilm, demonstrating powerful inhibitory effects against *H. pylori* biofilm [104]. However, its direct antibacterial activity against the bacteria themselves is relatively weak [105]. To address this limitation, Li et al. [106] and Arif et al. [107] developed RHL-chitosan hybrid nanoparticles for the delivery of clarithromycin or amoxicillin. The inclusion of antibiotics compensates for the relatively low antimicrobial activity of RHL alone. Both studies demonstrated strong therapeutic efficacy, achieving significant clearance of *H. pylori* biofilm beneath the mucus layer at the minimum inhibitory concentration, with clearance rates of 89 % and 99 %, respectively.

Building on these integrated strategies, Shen et al. [76] developed multifunctional self-assembling nanodrugs (BD/RHL NDs). These nanoparticles possess optimal size, a negative charge, and enhanced hydrophilicity, enabling them to successfully penetrate the mucus layer without interacting with mucin, driven by electrostatic and hydrophobic interactions. Both berberine and RHL, loaded into the nanoparticles, exhibit strong biofilm-disrupting properties. In vitro experiments demonstrated that BD/RHL NDs effectively degraded the EPS matrix and killed planktonic *H. pylori*, efficiently eradicating *H. pylori* biofilm. Berberine, a natural antimicrobial agent, not only disrupts biofilm but also inhibits microbial adhesion [108,109], blocking the critical step of biofilm reformation often associated with infection recurrence. This dual action significantly enhances the efficiency of complete *H. pylori* eradication.

*H. pylori* adheres to gastric epithelial cells by binding its BabA to the Lewis b antigen, promoting stable colonization and biofilm formation [110]. Targeting BabA is a promising strategy to inhibit adhesion and prevent biofilm regeneration. Zou et al. [111] developed antibiotic-free nanoparticles (FU/ML-LA/EB NPs) with a negatively charged FU coating. This design allows the nanoparticles to penetrate the gastric mucus layer and specifically inhibit BabA on *H. pylori*, competitively blocking adhesion and suppressing biofilm regeneration [112]. Furthermore, the FU coating interacts with multiple receptors on host cell membranes, such as scavenger receptors [113], enhancing cellular uptake of the FU/ML-LA/EB NPs. This activates AMPK to restore lysosomal acidification, thereby facilitating the effective degradation of intracellular *H. pylori*. Encapsulated urease inhibitor ebselen (EB) provides an effective antibacterial effect as an alternative to antibiotics [114]. Additionally, EB acts as an inhibitor of bis-(3′-5′) cyclic dimeric guanosine monophosphate, a bacterial second messenger with critical regulatory roles in the biofilm life cycle [115]. Thus, EB not only inhibits biofilm formation but also disrupts mature biofilms [116]. Moreover, the exceptional antioxidant properties of EB and linoleic acid (LA) [117,118] enable FU/ML-LA/EB NPs to alleviate excessive oxidative stress caused by *H. pylori*, reducing gastric mucosal damage and interrupting carcinogenic pathways. The antibiotic-free FU/ML-LA/EB NPs enhance *H. pylori* eradication rates by eradicating biofilms, eliminating intracellular bacteria, and alleviating oxidative stress, thereby preventing persistent infections. This approach represents a promising integrated strategy for *H. pylori* treatment. By reducing the development of antibiotic resistance, this antibiotic-free strategy also offers a novel multidimensional approach to combating *H. pylori* infections.

In summary, various therapeutic strategies targeting mucus penetration and biofilm disruption have significantly enhanced the eradication efficiency of *H. pylori* while reducing the likelihood of treatment failure and recurrent infections. These approaches not only improve drug bioavailability but also enable reduced dosages, thereby minimizing potential side effects.

### 3.3 Targeting virulence factors: urease and VacA

#### 3.3.1 Inhibition of urease activity

*H. pylori* produces urease, an enzyme that breaks down urea into ammonia and carbon dioxide, thereby neutralizing gastric acid and enabling the bacterium to survive in the stomach's acidic environment [16]. Urease, a key characteristic enzyme of *H. pylori*, was identified as a potential target for vaccine development as early as 1990, when researchers proposed the idea of creating a vaccine specifically targeting *H. pylori* urease [119] (Fig. 2c). However, oral vaccines were initially found to be ineffective due to the harsh and complex environment of the stomach, which rapidly degrades and inactivates the vaccine. Advances in nanotechnology have since made it possible to deliver oral vaccines more effectively, offering a promising solution to these challenges.

For instance, Tan et al. [120] developed an oral vaccine based on *H. pylori* urease and cholera toxin B subunit, delivered via HP55/PLGA nanoparticles. This dual-antigen and dual-adjuvant vaccine utilizes pH-dependent release characteristics, remaining stable in acidic environments and releasing the antigen in large quantities under neutral conditions. In animal models, the encapsulated vaccine nanoparticles successfully elicited high levels of systemic and local antigen-specific antibodies and a Th1/Th17-biased immune response, achieving a 43 % complete protection rate against *H. pylori*.

Similarly, Zhao et al. [121] designed a liposomal vaccine encapsulating recombinant fusion peptides of urease B epitopes and the cholera toxin B subunit. This vaccine demonstrated both prophylactic and therapeutic effects against *H. pylori* infection in BALB/c mice. Additionally, Zhang et al. [93] developed the R12/rUreB/PEG-Suc nanovaccine, containing recombinant urease subunit B, which significantly improved systemic and mucosal antibody levels in mice following oral immunization. This nanovaccine enhanced antigen-specific CD4^+^ T cell responses, boosted Th2 immune responses, and strongly induced IFN-*γ* secretion by antigen-specific T cells. Notably, the rUreB-loaded nanoparticles significantly reduced gastric colonization of *H. pylori*, demonstrating their ability to provide effective immune protection.

In conclusion, vaccines targeting urease show great potential for preventing and treating *H. pylori* infection. Anti-virulence therapy offers a theoretical advantage by selectively neutralizing bacterial pathogenicity through targeting virulence factors, achieving potent bactericidal effects [122]. However, the development of therapeutic strategies targeting *H. pylori* virulence factors remains limited. More detailed analyses of bacterial biofilm and virulence characteristics are needed to identify and optimize nanocarrier systems targeting specific virulence factors. Such approaches could maximize synergistic effects with conventional antibiotics while reducing the required antibiotic dosage.

#### 3.3.2 Targeting VacA-mediated pathogenesis

VacA is an 88 kDa soluble protein secreted by *H. pylori* into the extracellular space and serves as a significant virulence factor in the colonization and infection of the gastric mucosa. Despite its critical role, standard clinical treatments based on antibiotics often overlook this aspect [31].

Westmeier et al. [31,123] discovered that nanoparticles in simulated gastric fluid containing lipids and proteins can rapidly adsorb biomolecules from the gastric environment, forming a biomolecular corona. Liu et al. [124] developed poly (lactic-co-glycolic acid) (PLGA) nanoparticles coated with a lecithin bilayer (Ver-PLGA@Lecithin). Their experiments demonstrated that PLGA nanoparticles with a lecithin bilayer coating (PLGA@Lecithin) exhibited higher protein adsorption efficiency compared to PEGylated PLGA nanoparticles (PLGA@PEG). In vitro experiments showed that incubation with the model nanoparticles significantly reduced VacA content in the supernatant of *H. pylori* cultures by more than 80 %. Additionally, after 12 h of co-incubation, the cytotoxicity of the supernatant was reduced, and the survival rate of human gastric adenocarcinoma epithelial cells was notably increased. However, in animal models, utilizing the protein corona formation mechanism to adsorb VacA did not yield additional bactericidal effects. This suggests that therapies targeting VacA should prioritize alleviating *H. pylori*-induced gastric mucosal damage while incorporating simultaneous antibacterial treatment.

### 3.4 Metabolic interference: regulation of reactive oxygen species

#### 3.4.1 Reactive oxygen species regulation in bacterial metabolism

In recent years, dynamic therapy utilizing in situ-generated reactive oxygen species (ROS) to eliminate target cells has garnered significant research attention. ROS can oxidize various cellular components, including nucleic acids, proteins, and lipids, thereby disrupting bacterial metabolism [125] (Fig. 2d). This approach not only eradicates drug-resistant bacteria [126] but also helps delay the emergence of bacterial resistance [127,128].

Chakraborti et al. [129] designed and synthesized ZnO-polyethylenimine nanoparticles (ZnO-PEI NPs), which primarily mediate bacterial cell damage by generating ROS in situ. The PEI coating enables the nanoparticles to specifically target the LPS in the outer membrane of Gram-negative bacteria, facilitating nanoparticle internalization into bacterial cells. Additionally, this coating enhances the intrinsic antibacterial activity of the nanoparticles and increases ROS production. In vitro experiments revealed that, under ROS-mediated oxidative stress, *H. pylori* underwent a morphological transformation from rod-shaped to coccoid. This was accompanied by a rapid decrease in the levels of 16S rRNA and 23S rRNA, indicating significant bacterial stress and damage.

Nanozymes, with their superior peroxidase and oxidase-like properties, mimic the functions of natural enzymes and catalyze the production of highly toxic ROS [130]. As a result, nanozymes have emerged as a new generation of antimicrobial agents in antibacterial therapies [131]. Zhang et al. [132] developed a pH-responsive graphene nanozyme, PtCo@Graphene (PtCo@G), to selectively treat *H. pylori* infection. This nanozyme comprises PtCo nanocrystals isolated by functionalized graphene, which maintains excellent stability under harsh conditions. Under gastric acid conditions, the oxidase-like activity of PtCo@G is activated, catalyzing the production of large amounts of ROS and demonstrating bactericidal efficacy comparable to standard first-line triple therapy. To enhance targeting specificity, C18-PEGn-phenylboronic acid molecules are conjugated to PtCo@G, enabling precise targeting of *H. pylori*. This pH-responsive and targeting mechanism minimizes side effects on normal tissues and commensal bacteria. Interestingly, the Co element and graphene shell in PtCo@G@CPB facilitate magnetic resonance imaging (MRI) and Raman imaging, allowing the in vivo distribution of the nanomaterial to be detected without additional labeling molecules.

**Fig. 3:**
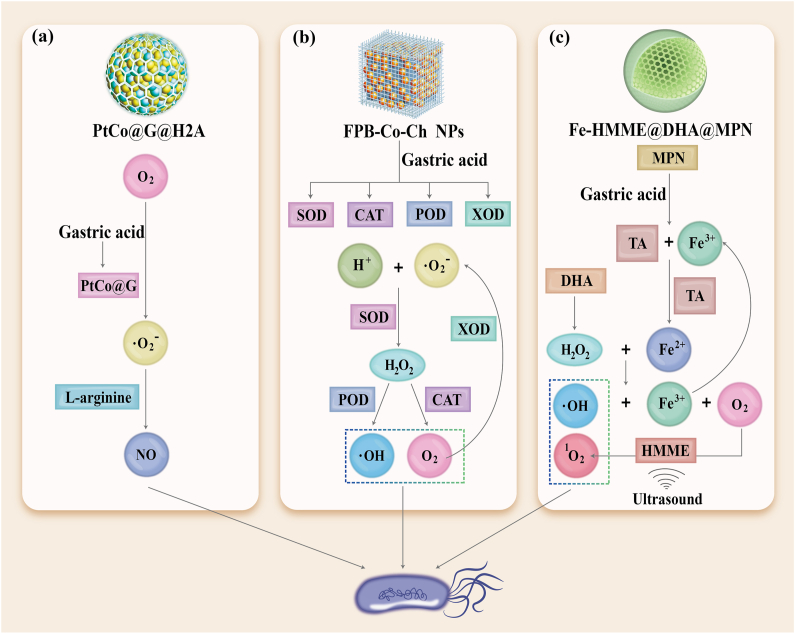
Cascade reaction design to enhance nanozyme-mediated ROS therapy against *Helicobacter pylori*. (a) The PtCo@G, activated by gastric acid, mediates a cascade reaction to generate NO for the eradication of *H. pylori*. (b) The FPB-Co-Ch NPs, activated by gastric acid, displays oxidase- (OXD), superoxide dismutase- (SOD), peroxidase- (POD), and catalase (CAT) mimicking activities, which mediate a cascade reaction to eliminate *H. pylori*. (c) Under ultrasonic treatment, the nanogenerator Fe-HMME@DHA@MPN employs a combination of sonodynamic therapy and chemodynamic therapy to treat *H. pylori* infection. PtCo@G@H2A: PtCo@Graphene@Heme-2(L-arginine); FPB NPs: ferrocenedoped Prussian blue analog; Co: Co_3_O_4_; Ch: chitosan; HMME: hematoporphyrin monomethyl ether; MPN: metal-polyphenol network; TA: tannic acid; DHA: dihydroartemisinin. (For interpretation of the references to colour in this figure legend, the reader is referred to the Web version of this article.)

ROS are a common strategy for treating antibiotic-resistant bacteria [133,134,135], but their short lifespan (3.5 μs) and limited diffusion distance (a few hundred nanometers) restrict their antibacterial efficacy. In contrast, nitric oxide (NO) has a longer lifespan (a few seconds) and a greater diffusion distance (100 μm), allowing it to better interact with bacteria and disrupt them by damaging proteins on the cell membrane and inhibiting DNA repair [136,137]. Moreover, NO can react with ROS to generate peroxynitrite anion, which exacerbates lipid peroxidation and damages the cell membrane [138,139]. Based on this, Deng et al. [140] developed a dual-targeted pH-responsive cascade catalytic nanozyme (PtCo@G@H2A) modified with heme-2(L-arginine) (H2A) to release NO in the highly acidic environment of the stomach for *H. pylori* treatment (Fig. 3a). PtCo@G@H2A undergoes a cascade reaction in gastric acid, first catalyzing ROS production and then oxidizing L-arginine in H2A to generate NO, achieving effective treatment of *H. pylori*. Additionally, this nanomaterial combines dual-targeting strategies to provide precise, efficient, and stable targeting. Heme specifically targets *H. pylori* by supplying the iron required for its survival, while a charge-reversal-mediated targeting strategy enhances targeting in the highly acidic gastric environment. In acidic conditions, the nanomaterial carries a positive charge, boosting its targeting effect in the stomach, while in neutral conditions, it carries a negative charge to minimize side effects on gut microbiota.

The design of cascade reactions through multiple steps can effectively enhance the generation of ROS, thereby maximizing the bactericidal effect while ensuring precise control over the reaction. Tong et al. [141] reported a nanometer-scale core-shell oxygen-generating system (FPB-Co-Ch NPs) that exhibits multifunctional, cascade-like nanozyme activities with pH-responsive properties (Fig. 3b). The system employs a novel iron-doped Prussian blue analogue (FPB NPs) as the core, with cobalt oxide nanoparticles (Co_3_O_4_ NPs) modified on its surface (FPB-Co NPs). Finally, the structure is coated with chitosan (FPB-Co-Ch NPs), which provides biocompatibility and bioadhesive properties. In the gastric environment, it mimics the activities of oxidase, superoxide dismutase, peroxidase, and catalase. These enzymatic activities facilitate the conversion of superoxide anions (•O_2_^−^) and hydrogen peroxide (H_2_O_2_) into oxygen, thereby contributing to the eradication of *H. pylori* in the stomach. Meanwhile, the nanozyme scavenges excess oxygen radicals, modulates the inflammatory response, and repairs damaged gastric mucosa. Under the neutral conditions of the intestine, the multifunctional nanozyme activity is suppressed, maintaining the balance of the gut microbiota. Additionally, the intrinsic magnetic resonance imaging properties of FPB-Co-Ch NPs enable the tracking of their biodistribution without the need for additional labeling molecules.

Photodynamic therapy (PDT) has been employed as a classic physical antibacterial method to treat bacterial infections over the past few decades [142,143,144]. PDT relies on the interaction between a laser and a photosensitizer to generate large amounts of ROS, which mediate bacterial cell death. Unlike antibiotic drugs, phototherapy does not require the internalization of the photosensitizer into cells [145], thereby reducing the risk of developing antibiotic resistance [133,[146], [147], [148]].

Qiao et al. [149] designed and developed a mucus-penetrating phototherapeutic nanomedicine (RLs@T780TG) for the treatment of multidrug-resistant *H. pylori* infection. RLs@T780TG consists of the near-infrared photosensitizer T780T-Gu and the anionic component rhamnolipids (RLs). Under near-infrared laser irradiation, the photosensitizer T780T converts light energy into heat and ROS, leading to protein denaturation and cell membrane disruption, thereby killing *H. pylori*. The positively charged guanidinium (Gu) groups enable RLs@T780TG to target *H. pylori* via electrostatic interactions while maintaining superior photostability. In vivo studies demonstrated that RLs@T780TG phototherapy achieved significant clearance of *H. pylori*, notable alleviation of gastric lesions, and minimal impact on intestinal microbiota balance compared to antibiotic treatments.

However, PDT is typically limited by the depth of light penetration, significantly restricting its application in the treatment of deep-seated diseases [150]. Leveraging the non-invasive and localized irradiation advantages of ultrasound, sonodynamic therapy (SDT) has gradually emerged as an alternative to PDT [151,152,153].

Wang et al. [154] developed a monoclonal antibody-conjugated liposome loaded with indocyanine green (ICG) (HpAb-LiP-ICG) for targeted in vivo photoacoustic imaging-guided SDT to treat *H. pylori* infection. The sonosensitizer ICG, which exhibits excellent biocompatibility, was approved by the FDA for clinical trials as early as 1956. Photoacoustic imaging can effectively penetrate deep tissues, enhancing the efficacy of SDT. Under ultrasound irradiation, ICG is activated to produce singlet oxygen, which selectively kills *H. pylori* by targeting the bacterial membrane via the specific binding of HpAb, without causing unintended toxicity to normal cells. Additionally, the photoacoustic signal allows for visualization of SDT in bacterial infections and precise observation of the nanomedicine's distribution within the body.

Similarly, Liu et al. [124] utilized poly (lactic-co-glycolic acid) (PLGA) nanoparticles coated with a lecithin bilayer (Ver-PLGA@Lecithin) preloaded with verteporfin to treat *H. pylori* infection. Verteporfin, like indocyanine green, is a sonosensitizer with excellent biocompatibility. The ultrasound output required for this therapy falls within the maximum safety limits set by ultrasound medical devices, indicating strong potential for clinical translation. In a female mouse model with gastric *H. pylori* infection, the combination of ultrasound exposure (0.5 W/cm^2^ for 10 min) applied to the stomach skin significantly reduced *H. pylori* infection, demonstrating effects comparable to standard triple therapy. Notably, unlike antibiotic treatments, SDT did not negatively impact the gut microbiota. The only significant effect observed was the upregulation of Lactobacillus, highlighting SDT's potential as a safer alternative to conventional therapies.

Bacterial infection sites, particularly biofilm-associated infections, are often hypoxic, limiting singlet oxygen production and reducing the efficacy of SDT. Enhancing oxygen availability or ROS generation can significantly improve SDT outcomes [135]. Chemical dynamic therapy (CDT), based on Fenton or Fenton-like reactions, has gained attention for its ability to generate hydroxyl radicals (•OH) from H_2_O_2_ and metal ions like iron. Moreover, the conversion of H_2_O_2_ to oxygen via Fenton reactions can enhance SDT. Combining CDT and SDT provides a more potent antibacterial approach; however, the low H_2_O_2_ concentration in bacterial infection microenvironments limits the effectiveness of this combination [155,156,157,158]. Additional H_2_O_2_ sources are therefore required to enhance antibacterial efficacy. Yu et al. [135] developed a novel metal-organic hybrid nanomaterial (Fe-HMME@DHA@MPN) with pH-responsive peroxidase-like activity, enabling selective antibacterial effects against *H. pylori* infections (Fig. 3c). The nanogenerator features a metal-polyphenol network (MPN) composed of tannic acid (TA) and ferric ions, which disassembles in acidic gastric conditions. Encapsulated hematoporphyrin monomethyl ether (HMME), a porphyrin-based sonosensitizer, generates singlet oxygen under ultrasound stimulation to achieve SDT. Simultaneously, released ferric ions are reduced to ferrous ions, which react with H_2_O_2_ to perform CDT via Fenton reactions. The nanoparticles also carry dihydroartemisinin (DHA), providing an additional H_2_O_2_ source to sustain CDT. Oxygen produced through Fenton reactions further enhances SDT through positive feedback, maximizing the therapeutic efficacy of the combined approach. Importantly, the pH-responsive nature of MPN suppresses peroxidase-like activity under neutral intestinal conditions, reducing toxicity to gut microbiota. This strategy demonstrated strong in vivo efficacy, comparable to standard triple-antibiotic regimens, and offers a promising approach for treating *H. pylori* infections.

#### 3.4.2 Iron ion regulation in bacterial metabolism

Gallium ions (Ga^3+^) effectively disrupt bacterial metabolism by competitively inhibiting iron-dependent pathways. With ionic radii similar to Fe^3+^, Ga^3+^ enters bacteria via iron transporters, interfering with Fe^3+^-dependent processes such as redox reactions and DNA synthesis, thereby exhibiting broad-spectrum antimicrobial effects [159]. Based on this mechanism, Lin et al. [160] developed a bubble-driven Ga/Zn micromotor with excellent biocompatibility and biodegradability for active bacterial targeting. The micromotor is fabricated by asymmetrically coating zinc microparticles with gallium. Hydrogen bubbles generated from the zinc-acid reaction propel the micromotor in gastrointestinal fluids at speeds up to 382.3 mm/s. Gallium enhances the zinc-acid reaction via galvanic effects, efficiently generating active antimicrobial Ga^3+^ ions. This mechanism shifts Ga^3+^ diffusion from passive to active, addressing the low diffusion efficiency of traditional nanomedicines and enhancing antibacterial efficacy. Importantly, the Ga/Zn micromotor degrades completely in gastric acid after releasing Ga^3+^, leaving no harmful residues and ensuring excellent safety.

The strategy of using ROS to disrupt bacterial metabolism offers notable advantages and significant potential. ROS can simultaneously damage key cellular components, effectively disrupting bacterial processes and combating drug-resistant strains. Nanotechnology further enhances targeted ROS generation, improving therapeutic efficacy while minimizing harm to healthy cells and the microbiota. However, challenges remain, including difficulties in precisely controlling ROS generation and the potential for damage to surrounding tissues, particularly in deeper infection where light penetration is limited. Future research aims to refine ROS release mechanisms and improve targeting specificity to reduce side effects. With advancements in nanotechnology and smart materials, ROS-based therapies are anticipated to achieve greater precision and effectiveness. Additionally, integration with imaging technologies may enable real-time monitoring, facilitating their translation into clinical applications.

### 3.5 Direct bactericidal mechanisms: membrane disruption and energy-based therapies

#### 3.5.1 Disruption of bacterial cell membranes

*H. pylori* infection is one of the most common chronic bacterial infections in humans, affecting over 50 % of the global population. The current therapeutic strategy for *H. pylori* primarily relies on antibiotic regimens. However, the increasing prevalence of antibiotic resistance and the recurrence of infections underscore the urgent need for alternative eradication strategies [161,162]. Various metal nanoparticles have demonstrated inherent antimicrobial properties, capable of disrupting bacterial cell membrane integrity, leading to the leakage of bacterial contents and, consequently, the direct killing of bacteria [73,163] (Fig. 2e). At low concentrations, metal nanoparticles are generally safe for human cells but lethal to bacteria and viruses, inducing cell damage through multiple mechanisms. These mechanisms make it difficult for bacteria to develop resistance [164,165,166].

Among metals, silver has been one of the earliest used in medicine due to its potent antimicrobial activity [167,168]. Camargo et al. [169] synthesized a nanoparticle-based drug (PN1) derived from a silver ion complex, [Ag(PhTSC *·* HCl)_2_](NO_3_) *·* H_2_O (Compound 1). PN1 exhibited high encapsulation efficiency, enhancing both the stability of silver ions and the controlled release of Compound 1. The silver ions in this system function by creating pores in the bacterial cell wall, leading to the leakage of cellular contents, disrupting bacterial metabolism, and ultimately killing *H. pylori* [170,171]. Additionally, the encapsulation within the nanoparticle carrier significantly reduced the compound's toxicity and lowered its minimum inhibitory concentration (MIC). Importantly, no toxicity was observed in *Tenebrio molitor* models treated with the nanoparticle drug.

Additionally, zinc ions exhibit antibacterial activity and can significantly inhibit urease activity, thereby accelerating the action of gastric acid against bacteria and effectively eliminating *H. pylori* [172]. Zhang et al. [172] reported a pH-responsive hydrogen-generating nanoparticle system, Pd(H)@ZIF-8, encapsulated in an ascorbyl palmitate (AP) hydrogel. The Pd nanoparticles, enclosed within the zeolite imidazolate framework structure (ZIF-8) framework, release hydrogen upon exposure to gastric acid. Through the targeted action of AP, Pd(H)@ZIF-8 anchors at the infection site, where it releases zinc ions (Zn^2+^) and hydrogen gas in response to gastric acid. The hydrogen gas disrupts the permeability of *H. pylori* cell membranes, facilitating the entry of Zn^2+^ into the cells. This promotes the leakage of cellular contents and interferes with the bacterium's metabolism. Additionally, the inhibition of urease by zinc ions enhances the action of gastric acid on the bacteria, effectively eradicating *H. pylori*. Interestingly, the released hydrogen gas regulates the secretion of inflammatory factors in macrophages, suppressing excessive inflammatory responses while scavenging excess ROS to alleviate oxidative stress-induced damage to gastric epithelial cells. Furthermore, it upregulates mucosal repair proteins, promoting the restoration of damaged gastric mucosa.

Copper ions (Cu^2+^) can disrupt bacterial cell membranes and effectively inhibit *H. pylori* urease activity, achieving efficient antibacterial effects [173]. Additionally, low concentrations of Cu^2+^ play a critical role in tissue regeneration by promoting angiogenesis and collagen deposition, facilitating gastric mucosal repair and preventing reinvasion by *H. pylori* after eradication [174,175,176,177]. Lai et al. [173] developed a multifunctional, antibiotic-free platform based on the copper-organic framework (HKUST-1). This platform, encapsulated in a lipid layer of phosphatidic acid (PA), RHL, and cholesterol, is further coated with chitosan and loaded into an AP hydrogel, forming AP@CS@Lip@HKUST-1. The negatively charged AP targets *H. pylori*-induced inflammatory sites via electrostatic attraction, where matrix metalloproteinases (MMPs) release CS-coated nanoparticles. The slow release of Cu^2+^ from HKUST-1 ensures antibacterial and mucosal repair efficacy while minimizing cytotoxicity [174,178]. PA enhances lysosomal acidification and autophagy, aiding in the clearance of intracellular *H. pylori*. Meanwhile, RHL disrupts biofilms, enabling the platform to eradicate planktonic, intracellular, and biofilm-associated *H. pylori*. This antibiotic-free approach effectively eliminates *H. pylori*, promotes gastric mucosal repair, and preserves intestinal microbiota homeostasis.

Additionally, gold nanoparticles (AuNPs) can induce cellular damage in mammalian cells through nonspecific mechanisms, including the induction of necrosis, apoptosis [179,180], oxidative stress, inflammation, DNA damage, and alterations in gene expression [181]. These effects highlight their significant potential as antimicrobial agents. Gopinath et al. [182] recently reported a size-dependent antibacterial effect of AuNPs against *H. pylori*. Gold nanoparticles with average sizes of 7 nm and 55 nm, synthesized at room temperature, exhibited antibacterial activity against multidrug-resistant clinical strains of *H. pylori*, with the larger nanoparticles demonstrating superior antibacterial efficacy. Furthermore, their MICs did not exhibit cytotoxicity towards AGS cells.

#### 3.5.2 Energy-based therapeutic approaches for bacterial eradication

The physical photothermal effect of AuNPs further enhances their antibacterial efficacy [148]. This photothermal mechanism offers the advantage of avoiding the development of bacterial antibiotic resistance in clinical applications. Zhi et al. [148] developed polyclonal antibody-targeted gold nanostar@*H. pylori*-antibody nanoprobes (GNS@Ab) for the in vivo treatment and diagnosis of *H. pylori*. Under near-infrared (NIR) laser irradiation, the photothermal effect generated by the GNS@Ab nanoprobes successfully eradicated *H. pylori*, including antibiotic-resistant strains, in the stomach. Additionally, *H. pylori* in the stomach was visualized using photoacoustic imaging. Importantly, within therapeutic dosage levels, oral administration of the GNS@Ab nanoprobes did not disrupt the balance of the gut microbiota, underscoring their potential as a safe and effective treatment for *H. pylori*.

In summary, metal nanoparticles offer significant advantages in directly killing bacteria by inducing cellular damage through multiple mechanisms. Unlike traditional antibiotics, metal nanoparticles can bypass common resistance mechanisms, making them particularly effective against antibiotic-resistant strains. Photothermal therapy (PTT), which utilizes heat to physically kill bacteria, represents another promising treatment strategy. Combining these two approaches further enhances therapeutic efficiency. However, several challenges must be addressed before these technologies can be widely adopted in clinical practice. A major concern is the potential toxicity of metal nanoparticles to human cells at high concentrations or during prolonged exposure. For energy-based therapies, precise targeting and minimizing damage to surrounding tissues remain critical hurdles. Nanoparticle-based and energy-driven antibacterial therapies hold substantial potential for clinical translation. With continued research into optimizing their safety, dosing, and targeting mechanisms, these technologies may provide effective alternatives to antibiotics for treating multidrug-resistant bacterial infections.

### 3.6 Microecological modulation by nanomaterials: direct and assisted regulations

The gastrointestinal tract is one of the most microbiota-rich organs in the human body, where the microbial communities interact closely to form a unique and complex ecological system. A healthy gastrointestinal microbiome is not only essential for digestion and immune regulation but also plays a crucial role in controlling the colonization of exogenous and opportunistic pathogens [183,184]. As a result, increasing research has shown that strategies targeting pathogen elimination are no longer limited to directly targeting the pathogens themselves but instead focus on modulating the local microbiota composition, demonstrating unique potential in inhibiting pathogenic bacteria. Therapeutic strategies related to microbiota modulation, though rarely reported in the treatment of *H. pylori*, have been extensively studied in the management of harmful gut bacteria. For example, Furuichi et al. effectively controlled the ecological niche of a community composed of 18 symbiotic strains by regulating the availability of glucuronates, thereby restoring colonization resistance and alleviating gut inflammation in mice induced by *Klebsiella* and *Escherichia* species [185]. Additionally, microbiota modulation strategies have been widely adopted in clinical practice, with fecal microbiota transplantation (FMT) now routinely used to treat severe, antibiotic-resistant *Clostridium difficile* infections [186,187]. These cases have shown better therapeutic efficacy compared to antibiotic treatments, effectively addressing the increasingly serious issue of antibiotic resistance. An increasing number of successful cases further validate the effectiveness of this approach.

This provides insight, suggesting that microbiota modulation is a potential therapeutic approach in counteracting *H. pylori* (Fig. 2f). In vitro experiments have shown that various microorganisms, such as *Lactobacillus plantarum* [188] and *Bifidobacterium* [189], can inhibit the growth of *H. pylori*, effectively regulate the composition of the gastrointestinal microbiota, safely and efficiently control *H. pylori* colonization, and prevent infection. Furthermore, microbiota regulation strategies can effectively address multidrug-resistant *H. pylori* and prevent the further development of *H. pylori* antibiotic resistance. Therefore, exploring and developing strategies for microbiota modulation is of particular importance.

#### 3.6.1 Probiotics-based nano-regulation

Traditional microbiota modulation strategies include dietary control [190], probiotic supplementation [191], FMT [192], and phage therapy [193]. Microbiota-derived therapies utilizing beneficial species and/or functions found in the human gastrointestinal have emerged as promising alternative strategies to alleviate pathogen colonization [194]. Probiotics, which are bacteria that produce lactic acid through the consumption of fermented foods, typically inhibit the growth of potential pathogens and the overgrowth of gastrointestinal bacteria by competing for nutrients and occupying niches in the gastrointestinal tract.

Due to the large surface area of the stomach and its dense mucus layer, orally administered probiotics face challenges in colonization and are often rapidly cleared due to gastric emptying. Fortunately, ongoing advancements in nanotechnology offer effective means to support probiotic colonization, providing opportunities for remote and precise modulation of biological systems. By effectively internalizing magnetic iron oxide nanoparticles, *Ruminococcus intestinalis* can promote its colonization in the gastrointestinal tract under the influence of a magnetic field [195]. To increase the adhesion time of delivery materials and improve the colonization rate of probiotics in the gastrointestinal tract, probiotic surface coatings can be designed based on specific colonization and therapeutic requirements. *Escherichia coli* Nissle 1917 produces a variety of antimicrobial substances that help inhibit the proliferation of harmful microorganisms and maintain a healthy balance of the gastrointestinal microbiota. It has been widely used in the treatment of various gastrointestinal disorders. As a candidate for restoring gastric microbial ecological stability, it is also considered a potential option for treating *H. pylori* infections. Luo et al. [196] chelated metal ions to enable the rapid formation of a nanocoating with ideal adhesion on the surface of EcN, thereby enhancing the colonization efficiency of EcN and successfully inhibiting the colonization of the pathogen *Salmonella Typhimurium*. Similarly, a lipid coating approach using platinum nanocatalysts (Pt-Lipid@EcN) improved the survival rate and colonization of EcN, while also improving gastrointestinal barrier damage [197]. Compared to single-strain probiotics, multi-strain probiotic supplements integrate different mechanisms, offering more powerful and comprehensive regulatory effects. Alkushi et al. [198] developed multi-strain probiotic nanoparticles (MSPNPs) containing *Bifidobacterium*, *Lactobacillus acidophilus*, and *Bacillus subtilis* with a dual coating of prebiotic chitosan and sodium alginate. This approach improved probiotic colonization efficiency, further enhancing the regulatory effects of MSPNPs by increasing the abundance of beneficial microorganisms while reducing the number of pathogens. Importantly, *Bifidobacterium* has been shown to antagonize *H. pylori*. Therefore, the design of surface coatings for probiotics using nanomaterials to improve colonization in the gastrointestinal tract holds great potential for *H. pylori* treatment.

Additionally, the acidic environment in the stomach and the presence of various digestive enzymes significantly affect the effective activity of probiotics. The long-term maintenance of probiotic viability, in order to achieve stable and sustained therapeutic effects, should be a key focus for researchers. Qiao et al. [199] developed EcN co-cultured with *Gluconacetobacter xylinus* in probiotic fermentation liquid. The bacterial cellulose (BC) synthesized by *G. xylinus* has a nanoscale fiber network structure, which endows the BC membrane with excellent biocompatibility and mechanical strength. This structure enables the membrane to protect EcN from damage caused by gastric acid, bile salts, and digestive enzymes, while controlling the release rate of EcN. Under specific conditions such as pH changes and enzymatic activity, the probiotics are gradually released, enhancing their stability and activity, thereby improving their therapeutic effectiveness.

#### 3.6.2 Intrinsic microecological regulation by nanomaterials

The unique adjustable properties of nanomaterials endow them with excellent adhesiveness and stability. This significantly enhances the colonization efficiency and activity of probiotics, making them an excellent biomaterial for supporting probiotic modulation of the gastric microbiota. Furthermore, increasing evidence suggests that nanomaterials themselves possess the ability to regulate the microbiota. Their tunable properties, as well as their capacity to carry and release active molecules, enable them to efficiently adapt to the gastric environment while exerting effective regulation. This provides a novel solution for gastric *H. pylori* infection microbiota therapy. Therefore, the application potential of nanomaterial-based microbiota regulation strategies in *H. pylori* treatment should not be overlooked and warrants further research and exploration.

Despite many studies suggesting that the regulatory mechanisms of inorganic nanomaterials in the gastrointestinal microbiota are not yet fully understood, some studies propose that inorganic materials may inhibit the growth of pathogenic bacteria by removing ROS and reactive nitrogen species, thereby modulating the microbiota [200,201]. AuNPs possess catalytic activity similar to catalase, enabling the breakdown of H_2_O_2_ and reducing oxidative stress. In an environment rich in amine groups provided by glycol chitosan (GC), AuNPs can further be transformed to exhibit superoxide dismutase-like activity. Kim et al. [202] combined AuNPs with glycyrrhetinic acid and coated them with GC to create a gold enzyme. This formulation significantly upregulated the abundance of beneficial microorganisms, including *Ackermansia*, *Lactobacillus*, and *Bacteroides*, while reducing the abundance of harmful bacteria such as *Enterococcus faecalis*.

Cerium oxide nanoparticles are commonly used in drug delivery and antioxidant therapy. Surface-coated cerium oxide nanoparticles (NP@ES100) with a ZIF-8 can restore the richness of the gastrointestinal microbiota and significantly increase the abundance of beneficial microorganisms from the *Enterobacteriaceae* family [203]. Further modifications of the nanoparticle size and the ratio of Ce^3+^ allow for the in situ growth of cerium oxide nanocrystals on a mixed membrane formed by red blood cell-derived vesicles and mesenchymal stem cell-derived exosomes. This mixed membrane, containing various growth factors, further enhances the abundance of probiotics, effectively regulating the balance of the microbiota [204].

Metal-based nanomaterials often lose their activity and antioxidant properties in vivo, which may limit their application in gastric microbiota modulation. However, negatively charged, metal-free melanogenic nanomaterials (MeNPs), known for their excellent biodegradability and biocompatibility, can overcome this issue. MeNPs significantly increase the relative abundance of *Bifidobacterium*, which has been shown to antagonize *H. pylori*. Meanwhile, the relative abundances of pathogenic bacteria such as *Bifidobacterium adolescentis*, *Alistipes*, *Bacteroides*, and *Escherichia coli*-*Shigella* are significantly reduced [205]. Gastric microbiota predominantly adhere to the gastric mucosal surface, and the gastric mucus layer, as a physical barrier, significantly influences the effectiveness of microbiota modulation therapies. Inspired by bacterial flagella morphology, Yin et al. [206] developed flagella-shaped poly (2-iodo-2-ethynylacetylene) (PIDA) nanofibers. PIDA adheres well to the gastric mucus layer, enabling effective microbiota modulation. Upon oral administration, PIDA significantly reduces the relative abundance ratio of *Clostridia* (phylum *Firmicutes*) to *Bacteroidetes* (phylum *Bacteroidetes*) (a ratio increase may be associated with inflammation), thereby restoring the microbiota composition to a healthier state.

Bilirubin mainly originates from the aging and breakdown of red blood cells, and not only exhibits antioxidant properties but also has the potential to regulate the microbiota. When bilirubin (BR) is combined with low molecular weight water-soluble chitosan nanoparticles (LMWC-BRNP), it can significantly reduce the relative abundance of *Turicibacter* in the microbiota associated with inflammation [207,208]. By combining the selective cyclooxygenase inhibitor Celecoxib with BR to form the dual-target nanomaterial Cel@HVGB, harmful bacterial proliferation is inhibited while the number of beneficial microorganisms (such as lactic acid bacteria) is increased [209]. Importantly, Zhou et al. [210] reported that *lactobacilli* not only inhibit the growth of *H. pylori*, but also suppress its virulence factors, thereby alleviating gastritis induced by *H. pylori* in mice. This suggests the potential application value of this nanomaterial in the treatment of *H. pylori* infection. To improve the bioavailability of bilirubin, Lee et al. [211] have combined it with hyaluronic acid (HA) to form a nanomedicine (HABN), enhancing its effects by targeting intestinal epithelial cells. HABN treatment can effectively increase the relative abundance of beneficial microorganisms, such as *Akkermansia muciniphila* (which helps protect the gastrointestinal barrier), *Clostridium XIVα* (which induces T regulatory cell production), and *lactobacilli*, improving gastrointestinal microbial species diversity and community richness.

Bacterial outer membrane vesicles (OMVs), as bio-derived nanomaterials, retain certain information and functions of their parent bacteria, which endows them with the potential to regulate microbial community homeostasis [212,213,214]. For example, Wang et al. [215] found that *A. muciniphila* OMVs significantly enhanced the abundance and diversity of the microbiome and modulated its dysbiosis by increasing the abundance of beneficial species and inhibiting the expansion of opportunistic pathogens. Similarly, Xu et al. [216] demonstrated that the membrane of EcN coated onto mesoporous silica nanoparticles not only enhanced adhesion to the mucus layer but also significantly increased bacterial diversity. This approach reduced the abundance of pathogenic bacteria such as *Mucispirillum schaedleri* and *Bacteroides vulgatus*, while increasing the abundance of short-chain fatty acid (SCFA)-producing bacteria like *Prevotellaceae UCG-001* and *Muribaculaceae*, shifting the microbiome profile toward an anti-inflammatory phenotype. Likewise, Li et al. [217] showed that coating EcN OMVs on manganese oxide nanomaterials effectively reshaped the pro-inflammatory microenvironment and microbiota. However, OMVs may undergo varying degrees of degradation or inactivation in the strong acidic and enzyme-rich environment of the stomach. The integration of nanotechnology is required to preserve their excellent regulatory effects in the stomach, thereby enhancing their potential for treating *H. pylori*.

Microbiota modulation strategies to inhibit the growth of pathogenic bacteria have gained increasing attention in recent years and have been widely applied in clinical settings, demonstrating excellent therapeutic effects. This treatment approach is based on the concept of modulation, which not only inhibits harmful bacteria but also promotes the growth of beneficial microorganisms. The aim is to reshape a healthier microbiota structure, control the colonization of pathogens, and effectively prevent re-infection. Additionally, microbiota modulation strategies can effectively mitigate the risk of antibiotic resistance development, avoid the side effects associated with antibiotics, and maintain normal digestive function. These strategies have shown great potential in the treatment of *H. pylori*. The advancement of nanoscience has not only provided novel regulatory tools but also effectively assisted traditional modulation strategies, offering opportunities for remote and precise biological system regulation with broad application prospects.

## 4 Nanomaterial-mediated drug resistance

**Fig. 4:**
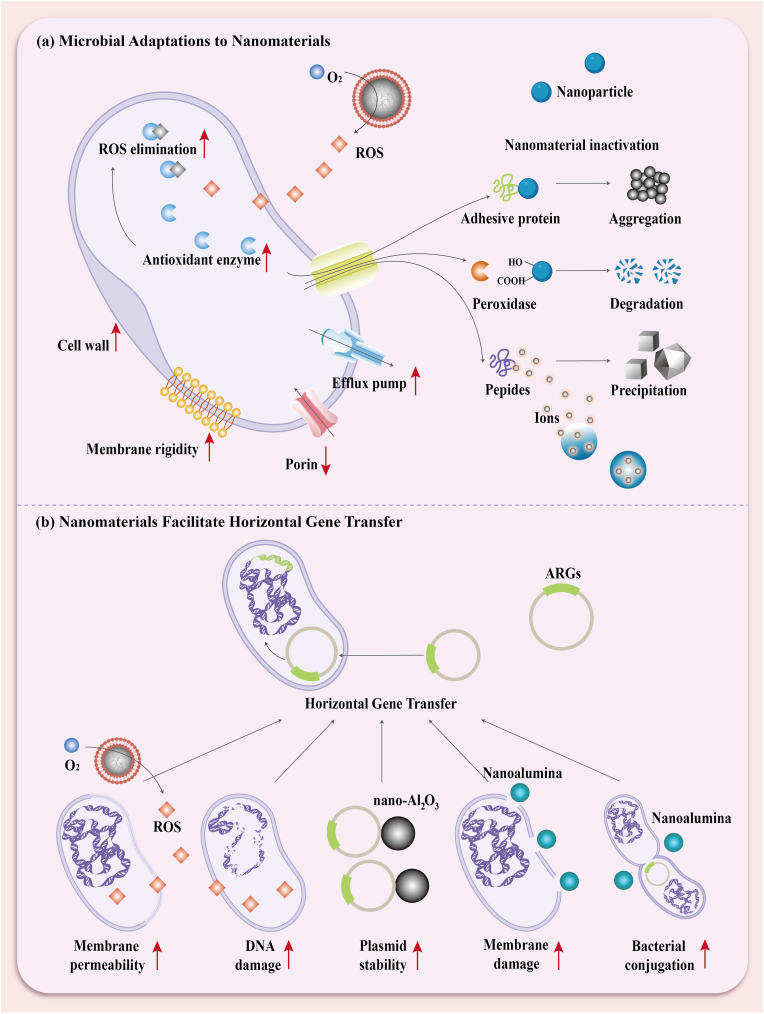
Nanomaterial-mediated drug resistance. (a) Microbial Adaptations to Nanomaterials. Bacteria resist metal-based nanomaterials by downregulating porins, overexpressing efflux pumps, and increasing cell wall thickness and membrane rigidity. They also upregulate antioxidant enzymes like superoxide dismutase and catalase to neutralize oxidative stress. Additionally, bacterial metabolism contributes to nanomaterial inactivation through aggregation, degradation, and ion precipitation. (b) Nanomaterials Facilitate Horizontal Gene Transfer. By increasing membrane permeability, inducing DNA damage, stabilizing plasmids, and promoting bacterial conjugation, nanomaterials enhance the transfer of antibiotic resistance genes (ARGs).

*H. pylori* resistance is becoming increasingly severe. Nanomaterials not only serve as efficient drug carriers, enhancing local drug concentrations to effectively kill resistant bacteria and inhibit further resistance development, but also offer bactericidal effects independent of antibiotics through diversified designs, providing a potential solution to the resistance problem. They show great promise and immense potential in the treatment of *H. pylori* infections. However, recent studies suggest that under sub-lethal selective pressure, bacteria may evolve resistance mechanisms to the bactericidal effects of nanomaterials that are similar to or distinct from those against traditional antibiotics (Fig. 4). This highlights the need to consider the risk of nanomaterial-induced resistance, even when nanomedicines are considered a highly advantageous strategy for combating resistant *H. pylori*. In the design of nanomedicines against *H. pylori*, researchers should promptly address and explore the dual aspects of “antimicrobial nanotechnology” to more effectively leverage nanotechnology for efficient sterilization while resolving the resistance issue.

### 4.1 Microbial adaptations to nanomaterials

When nanomedicines directly target bacteria through mechanisms similar to those of conventional drugs, similar resistance issues may arise (Fig. 4a). For example, metal nanoparticles can effectively kill antibiotic-resistant *H. pylori* by physically disrupting the bacterial cell membrane. However, some studies suggest that bacteria can develop resistance to metal-based nanomaterials by downregulating porin proteins to reduce ion permeability, or by overexpressing efflux pump systems to actively expel ions. A study by Radzig et al. [218] found that mutant *E. coli*, lacking OmpF/OmpC, exhibited reduced expression of porin proteins and impaired outer membrane permeability, which limited the intracellular uptake of silver ions. Moreover, Stabryla et al. [219] discovered that *E. coli* K-12 MG1655 cells developed genetic resistance to silver nanoparticles through mutations in CusS, a copper/silver ion sensor that drives the expression of the CusCBA efflux system to mitigate metal toxicity [220,221,222]. Furthermore, bacteria can resist nanomaterial penetration by remodeling their cell membrane structure, including membrane proteins, lipids, lipopolysaccharides, and peptidoglycan [223,224]. For example, after repeated exposure to nanoscale zero-valent iron, *Pseudomonas* species exhibited a transformation in unsaturated fatty acid conformation from cis to trans, leading to increased membrane rigidity [225]. In addition to membrane restructuring, bacteria may alter their morphology, such as filamentation and increased cell wall thickness, to counteract the effects of metal nanomaterials like titanium dioxide and nickel-manganese-cobalt oxides [226,227].

Mutations in *H. pylori* that alter the function of NAD(P)H flavin nitroreductase (FrxA) and/or oxygen-tolerant NAD(P)H nitroreductase (RdxA) reduce or inhibit the enzymatic reduction of metronidazole, leading to the intracellular accumulation of an inactive prodrug [228,229,230,231,232]. Similarly, certain bacterial metabolic processes can precipitate metal ions, degrade nanoparticles, or induce aggregation/crystallization, resulting in the inactivation of antimicrobial nanomaterials. The precipitation of released metal ions serves as a crucial adaptation mechanism in bacteria exposed to metal-based nanomaterials [233,234]. Naik et al. [235] identified two specific peptides capable of precipitating Ag^+^ and forming face-centered cubic silver crystals. Similarly, *E. coli* secretes adhesive proteins (such as flagellin A) to induce the aggregation of nanoparticles, thereby reducing the effective dosage of antimicrobial nanomaterials [236]. To alleviate the disruptive pressure of antimicrobial nanomaterials, *Trabusiella guamensis* releases peroxidase, which degrades multi-walled carbon nanotubes by catalyzing the oxidation of the tube surface into C = O and C− OH groups [237].

Compared to antibiotics, nanomaterials exhibit slower diffusion and tend to form aggregates, which facilitate bacterial behavioral adaptations. Long-term exposure to nanomaterials may select for highly motile bacteria capable of escaping nanomaterial-induced stress. For instance, hypermotile *E. coli* exhibited a more than twofold increase in tolerance to silver nanoparticles compared to non-motile strains [219]. *H. pylori* possesses flagellar structures that enable motility, highlighting the importance of considering the diffusion efficiency of nanomedicines in their design. This approach ensures efficient bacterial eradication and prevents adaptive selection of bacteria under non-lethal stress. Utilizing ROS to interfere with bacterial metabolism is an effective strategy against *H. pylori*. However, Pu et al. [238] and Yang et al. [233] found that bacterial cells may activate metabolic pathways to enhance the activity of antioxidant enzymes, such as superoxide dismutase and catalase, to eliminate ROS and mitigate oxidative damage induced by nanomaterials. Therefore, it is crucial to address issues such as low ROS concentration and diffusion efficiency through effective material design, such as cascade reactions, to enhance therapeutic efficacy.

### 4.2 Nanomaterials promote horizontal gene transfer

Unlike traditional antibiotic resistance, nanomedicines, due to their unique physicochemical properties, may introduce new interaction sites, thereby promoting the acquisition of antibiotic resistance genes (Fig. 4b). This can lead to the spread and transmission of resistance, resulting in the emergence of novel forms of resistance. Although the effectiveness of nanomaterials in directly delivering antibiotic resistance genes (ARGs) into bacterial cells remains questionable, they can serve as adsorption and immobilization surfaces for ARGs, increasing their interaction with bacteria or mediating interactions between antibiotics, heavy metal ions, and bacterial cells, thereby inducing gene transfer pressure and enhancing the likelihood of bacterial HGT [239,240]. For example, Yu et al. [241] found that the propagation of ARGs induced by CeO_2_ could be attributed to the increased production of extracellular polymeric substances and enhanced intracellular contact. Additionally, Qiu et al. [242] demonstrated that nanoaluminum could induce membrane damage, significantly promote bacterial conjugation, and facilitate the horizontal transfer of the *RP4* plasmid. Similarly, Ding et al. [243] demonstrated that nano-alumina (nano-Al_2_O_3_) can enhance the transfer of various ARGs across different bacterial strains, including those carried by large plasmids. This effect is attributed to its ability to tightly bind with plasmids, thereby protecting them from degradation. Copper ions have the ability to disrupt bacterial cell membranes and inhibit the urease activity of *H. pylori*, demonstrating an effective potential for the eradication of *H. pylori* infection [173]. However, Zhang et al. [244] demonstrated that both copper oxide nanoparticles and copper ions can significantly enhance the conjugative transfer of plasmid-mediated ARGs across different bacterial genera. This effect is attributed to the excessive production of ROS, which increases cell membrane permeability. Additionally, the upregulation of genes associated with oxidative stress and DNA damage response further promotes the horizontal transfer of ARGs. Although most of the reported genetic changes associated with antibiotic resistance in *H. pylori* are mutations rather than gene acquisition or loss [245], *H. pylori* demonstrates a high level of adaptability to horizontal gene acquisition and loss processes [246,247]. Therefore, this gene transfer may significantly increase the probability of *H. pylori* acquiring resistance genes, ultimately leading to its resistance to clinical antibiotics. This gene transfer may significantly increase the probability of *H. pylori* acquiring resistance genes, ultimately leading to its resistance to clinical antibiotics. This further emphasizes the urgent need for further research into the antimicrobial mechanisms and long-term safety of nanomaterials designed for the treatment of *H. pylori* infections, in order to minimize the potential development of resistance.

This phenomenon suggests that nanomedicines in antibacterial applications may not only trigger traditional resistance mechanisms but also promote the spread of *H. pylori* resistance through mechanisms such as gene transfer. Therefore, when designing and applying nanomedicines, it is crucial to fully consider the potential risk of resistance spread, establish appropriate evaluation systems, and take measures to prevent and control the emergence of resistance. Resistant bacterial cells can rapidly mutate to evade attacks targeting specific pathways, but mutations affecting multiple independent biomolecules are rarely reported [248]. Nanomaterials with parallel interactions and multiple targets have shown characteristics that inhibit the evolution of resistance. Thus, when designing nanomedicines for *H. pylori*, engineered nanocomposites should be prioritized to diversify the interactions between nanoparticles and microbes, achieving a synergistic bactericidal effect. Furthermore, the presence of resistant *H. pylori* may also be attributed to sub-lethal doses of antibiotics remaining in the stomach. Therefore, antimicrobial nanomaterials should be designed with controlled release mechanisms to precisely interact with target bacteria and reduce their residual amounts in practical scenarios. In addition, enhancing targeting specificity is crucial to minimize transgene-mediated resistance gene transfer. Designing core-shell structures and surface modifications to prevent nanoparticle aggregation can improve stability and antibacterial persistence. The future application of antimicrobial nanomaterials must explore multi-target approaches and precise delivery technologies to effectively combat bacterial resistance. In the treatment of *H. pylori* infections, nanomaterials still hold significant promise in eliminating pathogens and preventing resistance evolution.

## 5 Conclusion and future perspective

*H. pylori* is a microaerophilic, Gram-negative, spiral-shaped bacterium that infects approximately 50 % of the global population [1,2,3]. Chronic infection with *H. pylori* is responsible for 78 % of gastric cancer cases, making it the strongest known risk factor for gastric cancer development [4,5,6]. Currently recommended antibiotic-based treatments fail in approximately 20 % of cases, primarily due to increasing *H. pylori* resistance to antibiotics and the low bioavailability of antibiotics in localized infections [7]. The growing prevalence of antibiotic-resistant strains and the high rate of treatment failure pose significant challenges to existing *H. pylori* treatment strategies.

In response, extensive research has focused on identifying effective strategies to combat *H. pylori* infection and antibiotic resistance, including the development of new drug combinations, prolonged treatment regimens, and susceptibility testing prior to treatment. However, the acidic gastric environment and pepsin degrade most antibiotics, while the gastric mucus layer, approximately 200 μm thick, creates a pH gradient from 1 to 2 to 7.32, further reducing antibiotic delivery efficiency. These factors complicate eradication regimens, significantly affecting patient adherence and increasing treatment costs. Furthermore, antibiotic-based approaches carry the risk of exacerbating antibiotic resistance. Therefore, improving drug delivery efficiency and developing therapeutic strategies with multimodal bactericidal mechanisms offer significant promise for enhancing *H. pylori* eradication rates, improving treatment outcomes, and delaying the progression of antibiotic resistance.

Lipid-based and chitosan nanoparticles are widely utilized drug delivery platforms designed to deliver antimicrobial agents to target bacterial cells through multivalent interactions, such as receptor-ligand binding, hydrophobic interactions, and electrostatic attraction. Nanotechnology enables the encapsulation of active drugs, allowing them to evade enzymatic degradation, enhance stability, improve the permeability of therapeutic agents, achieve controlled release, and ensure efficient cargo delivery.

To overcome entrapment by the gastric mucus layer, surface modifications rendering nanoparticles “mucus-inert”, combined with guidance by external magnetic forces, have demonstrated improved penetration through mucus. Biofilm, which protect bacteria and are a common cause of treatment failure and recurrent infection, pose significant challenges. Agents such as RHL and silver disrupt biofilm integrity by interacting with the extracellular matrix and mediating signaling or other mechanisms.

Targeting *H. pylori* virulence factors directly, urease-targeted oral nano-vaccines have shown the ability to provide effective immune protection. Additionally, nanoparticles that adsorb the virulence factor VacA can reduce damage to the gastric mucosa during antibacterial treatment cycles.

Metal nanoparticles and nanozymes generate ROS in situ, disrupting *H. pylori*'s metabolic functions and effectively killing resistant strains while preventing the further development of antibiotic resistance. Photodynamic and sonodynamic therapies enhance the targeted and responsive release of ROS by nanoparticles conjugated with photosensitizers or sonosensitizers, further mitigating the adverse effects of antimicrobial therapy. Furthermore, the ability of metal nanoparticles to directly disrupt bacterial cell membranes significantly enhances bactericidal efficiency and prevents resistance progression. Gold nanoparticles combined with PTT utilize physical heat effects to kill bacteria and disrupt biofilm, significantly improving *H. pylori* eradication rates.

Novel drugs based on nanotechnology demonstrate excellent antibacterial activity; however, several limitations persist. Firstly, the complex preparation techniques and high production costs associated with nanoparticle drug carriers restrict their widespread promotion and application. Additionally, the combination of nanotechnology strategies with PDT or SDT is hindered by inefficient energy-to-effect conversion. Excessive doses of infrared light or ultrasound can cause irreversible damage to human tissues. Furthermore, the potential toxicity and low degradability of metal nanoparticles present significant barriers to clinical translation. The stimulus-responsive release functions of nanoparticle platforms require more rigorous animal studies to prevent adverse reactions from premature drug exposure in complex physiological environments. Lastly, while antivirulence therapy theoretically holds promise for selectively neutralizing bacterial pathogenicity by targeting virulence factors, the development of therapeutic strategies aimed at *H. pylori* virulence factors remains highly limited.

Increasing evidence suggests that microbiota regulation strategies have unique potential to inhibit pathogenic bacteria, which also applies to the treatment of *H. pylori*. Through microbiota regulation, the abundance of beneficial species can be selectively increased, and the growth of pathogenic bacteria can be suppressed, thereby reshaping the microbial community structure and transforming the microbiome into a more stable and healthy phenotype. Nanomaterials, with their special adjustable properties, can interact broadly with hosts or bacteria, enabling direct or assisted modulation of the microbiota. In terms of assisted regulation, nanomaterials significantly enhance the colonization efficiency of probiotics and effectively maintain their activity, leading to long-term stable therapeutic effects. Additionally, nanomaterials loaded with active ingredients or those with pro-oxidative properties can also efficiently regulate the microbiota. Therefore, using nanomaterials to modulate the gastric microbiota to antagonize *H. pylori* is a highly promising therapeutic strategy. It not only effectively inhibits the growth of *H. pylori* and prevents its recurrent infection but also significantly reduces the risk of further antibiotic resistance development.

In response to the growing issue of *H. pylori* resistance, nanomedicines have garnered widespread attention due to their bactericidal mechanisms independent of antibiotics and their potent antimicrobial effects. However, bacterial resistance is no longer limited to antibiotic resistance. Similar to traditional resistance mechanisms, under non-lethal selective pressure from nanomedicines, bacteria can evolve adaptive mechanisms to counteract these treatments, including mutations in efflux systems, inhibition of Omp family proteins, HGT, flagellar protein production, and cell membrane remodeling. Additionally, unlike traditional antibiotic resistance mechanisms, nanomaterials may also mediate interactions between antibiotics, heavy metal ions, and bacterial cells, thereby promoting HGT and acquiring cross-resistance to antibiotics. This highlights the need to recognize both the significant advantages of nanomedicine in treating *H. pylori* infections and the potential for nanomaterial-induced resistance. Researchers must continuously optimize designs by integrating multiple bactericidal mechanisms, developing controlled release systems, improving targeting accuracy, and enhancing the stability of nanomedicines. This approach aims to maximize antimicrobial efficacy while preventing the development of resistance to both antibiotics and nanomaterials.

In summary, this article outlines the major invasion and resistance mechanisms of *H. pylori*, as well as the current global status of resistance to this bacterium. It also reviews recent advancements in nanomedicine for the treatment of *H. pylori*, categorizing these approaches based on their design to target specific invasion or resistance mechanisms. The multifunctional modification sites on nanomaterials offer significant potential to maximize therapeutic efficacy while minimizing host toxicity. Nanomaterials not only serve as efficient drug delivery carriers but also integrate multiple bactericidal mechanisms, such as disrupting the cell membrane of *H. pylori*, generating ROS, and interacting with intracellular components. These features facilitate the effective eradication of *H. pylori* within cells and biofilm. Moreover, the regulation of the microbiota by the nanomaterials themselves and the supplemented probiotics has also demonstrated great potential in the application of combating *H. pylori*. Nanoparticle-based therapies for *H. pylori* hold substantial promise for future clinical applications and may provide viable solutions to the challenges posed by high levels of antibiotic resistance.

## CRediT authorship contribution statement

**Shi Wang:** Writing – original draft. **Hao Ding:** Resources. **Longsong Li:** Writing – review & editing. **Ruifang Zhao:** Writing – review & editing, Conceptualization. **Ningli Chai:** Writing – review & editing, Conceptualization.

## Declaration of competing interest

The authors declare that they have no known competing financial interests or personal relationships that could have appeared to influence the work reported in this paper.

## Data Availability

No data was used for the research described in the article.
